# On the Design of Ultra-High-Temperature Material Systems

**DOI:** 10.3390/ma18225203

**Published:** 2025-11-17

**Authors:** Panos Tsakiropoulos

**Affiliations:** Department of Materials Science and Engineering, Sir Robert Hadfield Building, The University of Sheffield, Mappin Street, Sheffield S1 3JD, UK; p.tsakiropoulos@sheffield.ac.uk

**Keywords:** alloy design, ultra-high-temperature material systems, refractory metal intermetallic composites, refractory complex concentrated alloys, refractory high entropy alloys, Nb-silicide-based alloys

## Abstract

In this paper an approach was proposed for the design of ultra-high-temperature material (UHTM) systems comprising a metallic UHTM substrate plus an environmental coating, based on past and on-going research in the author’s research group and in other research groups. The environmental coating, which was considered in order to discuss the design approach, was composed of a multi-material (multilayer) or functionally gradient bond coat, a thermally grown oxide, and a top coat. Issues that research must consider were identified. The complexity of UHTM systems was discussed, matters that design must take into account and the role of synergistic metallurgy were considered, and the importance of the environment was disclosed. The question of how the properties and behaviour of a UHTM system emerge was discussed.

## 1. Introduction

Since the 1950s, Ni-based superalloys have been the high-temperature materials (HTMs) of choice for aeroengines. This is ascribed to (a) their ability to operate to ≤0.88 homologous temperature (advanced superalloys melt at about 1350 °C, and exhibit significant strength to <1150 °C); (b) their alloying behaviour; (c) their ambient-, intermediate- and high-temperature mechanical properties; (d) their corrosion and oxidation properties; and (e) the ability of metallurgists to produce components using liquid or solid state processing. In high-pressure turbines (HPTs) in “state of the art” aeroengines, single-crystal (SX) Ni-based superalloy blades operate close to their highest application temperature limit, which is imposed by the melting temperature of Ni (1453 °C). Operation of HPTs with turbine entry temperatures (TETs) of up to <1600 °C has been possible because of advances in material processing, transition metal (TM), refractory metal (RM) and other metal (often referred to as “exotic,” e.g., Re, Ru) alloying additions, and internal cooling and environmental coatings (ECs) that allow substrate surface temperatures of up to <1150 °C [1,2].

To meet new environmental and performance targets in future aeroengines, the TETs should increase to <1850 °C, which would take substrate surface temperatures above the solidus temperatures of Ni-based superalloys. Thus, to move beyond the Ni superalloy era we need materials with capabilities exceeding those of Ni-based superalloys. The challenge we are faced with (or the problem to be solved) is to find new ultra-high-temperature materials (UHTMs), which could be ceramic, composite, or metallic advanced materials, with competences surpassing those of “state of the art” Ni-based superalloys. For the UHTM substrates, property targets show where the R&D road must take us.

The property targets for UHTM substrates, which have been set by industry, are as follows: (a) the creep strength should be greater than 170 MPa at a creep rate of 2 × 10^−8^ s^−1^ at 1200 °C, which assumes an alloy density ρ = 7 g/cm^3^ (for materials with different densities the required creep strength must be adjusted; for example, for materials with ρ = 9.5 g/cm^3^, the creep strength goal is 230 MPa at 1200 °C with a creep rate of 2 × 10^−8^ s^−1^); (b) the fracture toughness of critical components should be ≥20 MPa√m; and (c) the recession rate due to oxidation should be less than 0.25 μm/h at 1315 °C (this goal was derived from the requirement to achieve an oxidation life at 1315 °C of the 2nd generation SX Ni-based superalloys at 1150 °C) [1,2,3]. The toughness goal requires the new substrates to show some degree of metallic behaviour, to distinguish them from engineering ceramics or ultra-high-temperature ceramics.

The toughness goal necessitates the new substrates to have at least a small volume fraction of a ductile, metallic phase, and has given the advantage to metallic substrates [1,3,4,5]. The latter are based on, or make use of, RMs [4,5,6]. Owing to the anticipated capabilities of the new substrates at high temperatures, the new metallic UHTMs are referred to as metallic UHTMs. The latter are refractory metal intermetallic composites (RMICs), refractory metal complex concentrated alloys (RCCAs) and refractory metal high entropy alloys (RHEAs) [5,7] (see Abbreviations).

Similar to Ni-based superalloys, metallic UHTMs will need protection with environmental coatings [7]. The resulting UHTM system would thus be composed of a metallic UHTM substrate plus environmental coating. The design of the substrate cannot ignore the need for the EC. It is the opinion of the author that the priority of R&D should be the concurrent design and development of substrate and environmental coating, rather than the separate design and development of substrate(s) and then coating(s), which has been the case for components based on Ni-based superalloys. To the author’s knowledge, UHTM system property targets have not been set by industry. In our research group the target for UHTM systems is to achieve, with TET < 1850 °C, the performance of 2nd generation SX Ni-based superalloys with internal cooling and environmental coating in “state of the art” aeroengines. For the UHTM system there should be a strategic paradigm, i.e., a set of ideas to guide how to develop and implement strategy [6]. These are logically and topologically inseparable.

The history of alloy design and development (for example, see [2,8,9,10,11,12,13]) is an indivisible part of metallurgy itself, it is indispensable for its further development and for attracting others and giving content to the theories it contains at any particular moment. The philosopher Wittgenstein cautioned us that “science contains not only formulae and rules for their application but entire traditions” (attributed to Wittgenstein in [14]). We know from Kuhn (1) that “a paradigm is a tradition containing easily identifiable features side by side with tendencies and procedures that are not known but guide research in a subterranean way and are discovered only by contrast with other traditions” [14,15] and (2) that “different paradigms (a) use concepts that cannot be brought into the usual logical relations of inclusion, exclusion, overlap, (b) make us see things differently (research workers in different paradigms have not only different concepts, but also different perceptions) and (c) contain different methods intellectual as well as physical instruments of research” [14,15].

The solution to the said problem (see the second paragraph) cuts right across the borders of different subjects or disciplines [6]. “Science is an art” wrote the Nobel laureate G Thomson [16]. Metallurgical and materials engineers and other engineers and scientists are proceeding to formulate tentative solutions to the problem. The solutions, in the light of experimental evidence, which is interpreted in the light of theories [17,18] (see theory in Appendix A), are being subjected to discussion and criticism. Because of the latter and because of theories that run into difficulties, and against a background of information/data and knowledge (see information and knowledge in Appendix A), new problems have arisen. Owing to the “problem” to be solved there is more than one problem to start with, with a multiplicity of tentative solutions to problems and many different criticisms raised.

It has become customary for engineers and scientists to seek solutions to problems via cooperation and discussion in terms of a common agenda or by working in a common framework (see framework in Appendix A) to which they have committed themselves. They use theories that are said to be commensurable (see commensurability in Appendix A). Many engineers and scientists give priority to mathematical formalism, tools and computation rules, and their applications for prediction purposes. The use of a specific framework helps, in part, to position their work in a field and its related concepts, theories and paradigms [15,19]. A rational discussion is thought to be impossible without an established framework (without the same standards (see standards in Appendix A) of “measurement”). However, the solution to specific problem(s) may require researchers to move to a new framework. Solution(s) to problem(s) are sometimes impossible without faith in speculative ideas. The choice of method(s) with which to solve a problem (see problem in Appendix A) depends on the choice of objective. The two ways of looking at the problem (with the existing and a new framework) could be considered by some as being inadequate, on the grounds that rational discussion would be impossible (not able to be compared or judged by the same measure or standard). Theories are said to be incommensurable (see incommensurability in Appendix A) if they are embedded in contrasting conceptual frameworks or paradigms. However, theories can be interpreted in different ways. They will be commensurable in some interpretations, incommensurable in others [17].

In a recent paper [6], I presented a personal perspective of the design and development of metallic UHTMs. In [6], I drew the alloy design “landscape” (see Figure 1 in [6]) of the alloy design methodology NICE [4] to demonstrate the significance of (a) synergy, entanglement and self-regulation for alloy design and development, and of (b) emergence (see [6], and Section 4 in [20]) for understanding the properties of phases in metallic UHTMs, for example see [4,5,6,20,21], and the properties of metallic UHTM substrates and UHTM systems that could be used beyond the Ni superalloy era [2,6]. Note that all methodologies have their limits. “Ernst Mach (see problem in Appendix A) examined methodological principles by showing how they aided or hindered scientists in the solution of concrete problems. Later, Einstein developed this procedure into a fine art” [14]. NICE describes a structured procedure for bringing about a certain goal (see Sections 4 and 5 in [6]), e.g., to discover new knowledge, to establish correlations between practice and results, such as between parameters (see below Section 4.1) or phases, between parameters and phases or properties and parameters, to clarify the structure of data and see phenomena in a new light, to use experimental data with the goal of finding relationships that can be used to make predictions, or to understand studied phenomena and use theories, say of solid solution strengthening or oxidation, to make calculations.

Additionally, in [6] I proposed the notion of synergistic metallurgy. I pointed out that synergistic metallurgy in partnership with chemical, mechanical, physical and process metallurgy, works together with the science and engineering disciplines and has the capacity to affect how materials are developed, produced and used, and change the world. Complexity (see complexity in Appendix A) characterises the behaviour of a UHTM system whose components interact in multiple ways and follow rules leading to emergent properties (see below, and [20,21]). The complexity of UHTM systems has common as well as distinct features compared with complex systems in general, for example see [22,23,24]. Complexity increases the number of emergent behaviours. Emergent behaviours and properties are characteristics that are also associated with complex systems [22,23,24]. Synergistic metallurgy helps deal with complexity but also increases complexity.

The aim of this paper is to propose an approach to the design of UHTM systems, and to discuss key issues to be considered by R&D. First, I shall describe a UHTM system that I shall use as the vehicle to set in motion my case, then I shall give further thoughts on alloy design and development, followed by a brief discussion of complexity in UHTMs and finally I shall consider the issues that I deem to be important for the design of a UHTM system. Explanatory notes for terms used in the paper are given in Appendix A. These notes are intended to help the reader to comprehend the approach that I propose for the design of a UHTM system.

## 2. UHTM System

**Figure 1:**
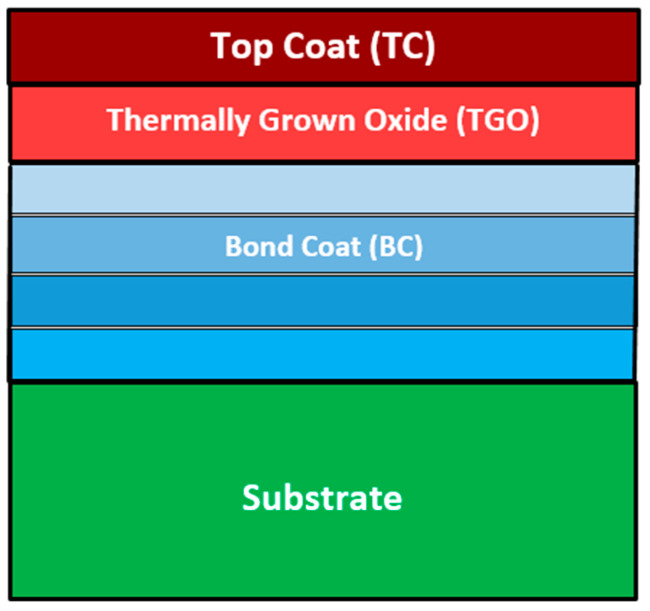
Schematic diagram of proposed UHTM system consisting of substrate and environmental coating (see text). The substrate would be a metallic UHTM, and the environmental coating will consist of three parts, namely a bond coat (BC), thermally grown oxide (TGO) and top coat (TC). The bond coat would be either multilayer (in the figure the BC is shown to consist of four layers), or functionally gradient material.

In our research group we have identified a specific UHTM system that deserves research to ascertain whether it would be suitable for an era beyond that of the Ni superalloy, namely a UHTM system consisting of a substrate plus an environmental coating (see Figure 1) in which the substrate is a metallic UHTM—such as an RMIC, RCCA, or RHEA (see Abbreviations, and [7])—and in which the environmental coating (EC) consists of a bond coat (BC), thermally grown oxide (TGO) and top coat (TC) [6,7]. The aforementioned UHTM system consists of the same parts (meaning substrate plus environmental coating) as the material systems, such as, for example, the aerofoils currently in use in HPTs in the Ni superalloy era of aeroengines. The parts of this specific UHTM system have different properties and have causal relevance.

There are differences regarding the components that could make up the parts of the UHTM system that is proposed for an era beyond that of the Ni superalloy. For example, regarding the environmental coating, the BC could be a multilayer material (a multi-material) consisting of “conventional” alloys or RMIC(s), RCCA(s), or RHEA(s), as shown schematically in Figure 1, which displays a BC consisting of four materials, or a functionally graded material, which could be a “conventional” alloy or an RMIC, RCCA, or RHEA [7,25]. The TGO should preferably be (i) α-Al_2_O_3_, or, failing that, could consist of (ii) alumina plus oxides that have prototype TiO_2_ (rutile), which may include niobates, e.g., CrNbO_4_ niobate (which, however, grows faster than alumina), or Ti_(1−x)_Nb_x_O_2_ compositions, e.g., TiNb_2_O_6.42_ that is resistant to fracture [4] or (iii) alumina, silica and (Ti_1-x-y_Cr_x_Nb_y_)O_2_ [26] (note that silica scales are volatile in water-vapour-loaded environments). The TC should be a ceramic material with capabilities beyond those of yttria-stabilised zirconia, and could be “conventional” or high entropy (HE) or complex concentrated (CC) ceramic(s).

The environmental coating of the proposed UHTM system should be required to provide the same protection to the metallic UHTM substrate as the current environment coatings do for Ni-based superalloy substrates, with the extra requirement to protect, to a feasible extent, the UHTM substrate from interstitial contamination while enabling the UHTM system to operate with turbine entry temperatures at least 150 °C higher than currently used in state of the art aeroengines. Oxidation resistance as well as resistance to interstitial contamination are essential requirements for the metallic UHTM substrate. Furthermore, resistance to pest oxidation [4,5] is a requirement for all metallic parts of the UHTM system. Problems linked with calcia-magnesia-alumino silicate (CMAS) [27] must also be addressed by the design of the EC, the selection of the top coat, and by the method(s) used for the manufacture of the UHTM system.

In the multi-material (layered) bond coat, the key issues are (i) the correct selection and correct sequence of layers and their mutual relations and (ii) whether the resultant effect (i.e., the bond coat) can be understood in terms of a harmonious layering or as a case where one or other of the layers dominates the others to give the bond coat the desirable behaviour in the environmental coating.

Next, I shall consider the selection of parts of the UHTM system. I shall draw attention to synergy, entanglement, and self-regulation, and then briefly comment on complexity and emergence. I shall revisit emergence and emergent properties in the last section of the paper.

## 3. Selection of Parts for the UHTM System

How can microstructures, which are formed within a metallic UHTM substrate or a UHTM system in a specific environment in time and space, be accounted for by metallurgy? Or more concisely rephrased “how does a metallic UHTM substrate or a UHTM system work in a specific environment?”

Any attempt to answer the above question depends (a) on how wide a field of view one takes, and (b) what questions one asks and attempts to answer. How the UHTM system to be studied was made is also very important. Progress regarding alloy design, alloy development and discovery of alloys that meet property goals depends on both (a) and (b). “One” here means an individual researcher, or a research team, or group(s) of national or international researchers/teams, or institutions providing funding, or institutions encouraging methodical learning.

The design of metallic UHTMs has proved more challenging than many had anticipated. With RHEAs and RCCAs the possibilities have expanded even further compared with RMICs, and alloy designers are confronted with new challenges. In our research group we have developed the alloy design methodology NICE [4,5,6,7]. The latter is central in the design and development of the said UHTM system.

Factors contributing to the difficulties faced by alloy designers include (i) the lack of data about, for example, economic indicators or environmental conditions that can inform alloy design [5,6], (ii) the lack of and disagreements about thermodynamic data [4,5], and (iii) the limited availability of resources worldwide (a) for scaling up alloy making and (b) for evaluating mechanical and environmental properties using specimens from large ingots [5]. In turn, (i) (ii) and (iii) have affected both the breadth and depth of experimental work, calculations and modelling.

Processability issues (including machining) do not seem to be a priority of current research. The majority of research on metallic UHTMs continues to use small arc melted buttons. Impressive properties have been reported for some alloys that were made as small arc melted buttons. Have these been reproduced with large ingots? Reproducibility of results using large ingots is a challenge that research on metallic UHTMs must address [5]. Calculations and modelling have hardly addressed multiphase metallic UHTMs or UHTM systems.

Many metallurgists (including the author) use the geographical metaphor (see metaphor in Appendix A) landscape to discuss alloy design and to show how the building blocks of an alloy are strung together (see Appendix A) [6,13].

Metaphors do real conceptual work. A methodology (see [6]) is reasonable and comprehensible only in its relation to a given problem situation. It can be rationally discussed only by discussing this relation. In the alloy design methodology NICE [4], the metaphor “landscape” facilitates new ways of thinking about alloy design and development, and the notion of synergistic metallurgy is key [6]. A significant part of the mission of NICE is to produce information and knowledge that can be a resource for metallurgical research on metallic UHTMs and UHTM systems.

How multiphase microstructures are produced to sustain alloys in a specific environment, to allow alloys to continue to function for a period of time in a specific environment, is not that simple. The metaphor “landscape” offers consolation. Metallurgical research over the past years has painted a richer and much more astonishing picture. The picture at times appears perplexing, relying on principles and processes of organisation (see organisation in Appendix A).

The ability or capacity of a metallic UHTM substrate or a UHTM system to do something (e.g., to withstand loads in a specific environment) or to act in a particular way (e.g., to be resilient, adaptive in a specific environment) depends on the phases that work together and are entwined [6] to generate the behaviour of the substrate or material system. For example, regarding metallic UHTM substrates, the bcc solid solution(s) benefits toughness and ductility but not oxidation resistance or creep; the tetragonal M_5_Si_3_ silicides (tI32, D8_m_, prototype W_5_Si_3_ or tI32, D8_l_, prototype Cr_5_B_3_) benefit strength and creep but not toughness and ductility, whereas C14 MCr_2_ Laves phases benefit oxidation resistance but not toughness; and A15 M_3_X (X = Al, Ge, Si, Sn) compounds benefit oxidation resistance (M is transition metal(s) (TMs)) [5,6].

According to NICE, the phases of a UHTM system will be in specific localities in parameter or phase maps [5,20,28,29]. Phases that form at interfaces between parts of the system over a course of time owing to internal (e.g., interdiffusion) and external (e.g., interaction with the environment) influences will also be in specific localities in parameter or phase maps [20,30,31].

The elements of the UHTM system will be in synergy and entangled in its in-service life [6]. For example, research has shown that, in RM(Nb)ICs and RCCAs/RM(Nb)ICs, (i) Sn suppresses tetragonal (tP32, prototype Ti_3_P) Nb_3_Si [32] and pest oxidation [33,34,35,36,37,38] while its partitioning behaviour is affected by other solutes [33,34,39,40,41,42,43]; (ii) Mo improves the creep [44] and oxidation resistance of the alloys [45], and the yield strength of bcc solid solutions [5] but its concentration in the latter depends on that of Ti [21]; (iii) Si concentration is key to the creep, high temperature strength, toughness and oxidation resistance of the alloys [1,3,5,46] but its concentration in bcc solid solutions depends on that of Mo or Mo + W [4,28,39], but not on Ta or Ta + W [42,47]; (iv) Ge partitions to the Nb_5_Si_3_ silicide rather than the bcc solid solution [48], suppresses pest oxidation and improves oxidation resistance of the alloys at high temperatures [49] and its partitioning behaviour is affected by other solutes [39,40,42,50]; (v) W partitions to the bcc solid solution where it has a strong effect on the concentration of Ti [39,51], and improves the yield strength of the bcc solid solution [5]; (vi) Ge and Sn together have a strong effect on the vol.% of bcc solid solution [39,40] and the partitioning of Al to the solid solution [21]; and (vii) Ge and Sn together with Al and Si control the contamination of Nb_5_Si_3_ with oxygen [21].

### 3.1. Synergy, Entanglement, Self-Regulation in the UHTM System

Research in our research group and in other research groups has shown that what the elements do collectively in a metallic UHTM substrate, i.e., in synergy [6], is key for the design of these advanced materials and UHTM systems. Most solute elements can accomplish very little alone, with the exception of (a) interstitials, e.g., oxygen, whose role becomes crucial as contamination progresses (i.e., as the concentration of interstitial(s) in the material (near its surface and in bulk) increases, e.g., see [52]), or (b) specific solutes that (i) suppress undesirable phases, e.g., Al [53], Sn [32], or (ii) stimulate the formation of phases that improve properties, e.g., Ge [49].

As a result of synergy [6], solute elements (including interstitials) form phases that can grow and transform. They are makers of meaningful relationships and generators of meaning (see meaning in Appendix A) (for example, phases produce meaning for a material by enabling it to achieve its objectives, for example, to meet property targets). The notion of meaning embeds and entangles the material or material system in its environment. The material system works in relation to its environment. Materials generate meaning as they evolve. Evolution can change the performance of material systems. The design of UHTM systems must take into account evolution. This is feasible with the alloy design methodology NICE (see Table 1 in [6]) and with synergistic metallurgy (see Section 7 in [6]).

On account of synergy, entanglement and self-regulation (see Appendix A), metallic UHTM substrates and UHTM systems could be designed to be versatile, adaptive (see adaptation in Appendix A) and robust. The *modus operandi* of NICE and synergistic metallurgy is to furnish a genuine understanding of how UHTM systems work.

We can think of synergy, entanglement and self-regulation acting as an agency (i.e., acting as a genuine cause of change not instantaneously but over periods of time). What goes on inside the alloy design landscape is influenced by what happens outside, e.g., the environment (see Figure 1 in [6]). In consequence of property targets for the system, the agency can change so as to fit a new or specific use, i.e., the agency can have adaptive strategies.

For design purposes, an alloy can be considered as a site of the movement of matter, energy and information (i.e., of the systematic imparting of data, information, knowledge and understanding). The alloy designer selects and then, via some processing method, e.g., melting and casting, brings together a number of elements to form, say, a metallic UHTM substrate to achieve specific target(s).

Synergy, entanglement and self-regulation, acting as an agency, guide the alloy designer using (in our case) the alloy design methodology NICE and its alloy design landscape to organise (see organisation in Appendix A) the microstructure in partnership with information, knowledge, meaning and targets (see Table 1 in [6], and below). In this way the properties of a metallic UHTM substrate or UHTM system emerge, see [20] and below.

Solute elements and phases in a UHTM system are mobilised towards achieving a property target. The relationships between solutes signify (see information in Appendix A) a dynamic, i.e., evolved capabilities. Synergy, entanglement and self-regulation, acting as an agency, guide the mobilisation. Key ingredients are data, information, knowledge and meaning (for example, environmental stimuli indicate what needs to be achieved by alloy designers if the metallic UHTM substrate were to survive in a specific environment). The latter is a prerequisite to achieving a property goal. The aforementioned and entropy changes, which accompany the material during its life, nurture the UHTM system.

It is my opinion that the design and development of UHTM systems must reflect on the notions of complexity and emergence in these materials. Complexity and emergence are briefly considered in the following section.

### 3.2. Complexity and Emergence in the UHTM System

Complexity is a word with rich connotations and is used in many ways both formally and informally. There is no unique definition of complexity. Though the word complex is used only for RCCAs (see Abbreviations), complexity is a characteristic feature of all metallic UHTMs and UHTM systems. A metallic UHTM or a UHTM system is complex not only of what it is and does but also because of what it goes on to do.

Complexity (see Appendix A) is concomitant with how the material or material system is made up and evolves, i.e., its structure. The parameters that control a complex system are important as well as the information content (but how do we define and measure information?). We should not expect to have a single universal measure of complexity. We are likely to have a measuring system used by a particular observer or practitioner/specialist in a particular context for a particular purpose (see emergence below). The complex material or material system has emergent properties [6,7,20,21] and behaviours. Complex materials or material systems can create a new structure. A new structure can emerge through different kinds of interaction.

Connectivity (and the interdependence that arises from the connectivity) is essential for a material system. As solutes and phases interact, complex behaviour(s) is (are) created and the solutes, phases and different parts of the material system become connected with each other. The quality and intensity of connectivity varies with time. When designing a UHTM system, owing to complexity, we must allow for uncertainty and predictability and the system evolving.

The emergence that arises from the connectivity and interactions in the material system constrains certain behaviours of the connected and interacting parts, and at the same time opens new possibilities. Connectivity and interactions make emergence a very dynamic process.

Complex material systems could learn, adapt, self-regulate, self-organise and change with time, and in this way complexity changes. This feature is linked with feedback (see feedback in Appendix A) within the material system, and the resulting change is not a property of the structure of the system per se but of how that structure changes. Thus, in terms of designing and developing a material system, the ability to learn and adapt is important. Synergy and entanglement are key for learning and adapting.

The interactions and dependencies between the solutes and phases, chemical heterogeneities (e.g., segregation), defects, and impurities (including interstitials from contamination) of a material system are conclusive for its complexity. The spatial and temporal scales of the material system are also important for its complexity. Furthermore, the strong influence that the structure and properties at the microscale have on the structure and properties at the macroscale increases the complexity of the system.

The interactions, dependencies and multi-scale effects make it difficult to understand and develop material systems. To understand and develop a material system, the question of how interconnected are the individual components of the system is more important than the number of its components. In the next section I shall discuss key issues that to my knowledge have not been considered by alloy designers, and which are key for the design and development of a UHTM system. In addition, I shall discuss how these issues are considered in NICE.

## 4. Issues for the Design and Development of the UHTM System

In this section I wish to expand on important challenges about the design of a UHTM system. I shall use as an example the UHTM system that was described in Section 2 with an RM(Nb)IC, RM(Nb)IC/RCCA, or RCCA/RM(Nb)IC as the substrate, and RCCA(s) or RHEA(s) as BC(s). Different approaches could be used by researchers and material developers to design and develop UHTM systems. I shall refer to the alloy design methodology NICE [4] and its alloy design landscape [6], i.e., the tools that we use in our research group to design and develop UHTM systems, to discuss interaction and connectivity, relationships of differentiation and similarity, self-organising, information and knowledge, learning and adaptation, and meaning and emergence. For explanatory notes for some of these terms please refer to Appendix A. The reader will also find [6] useful.

### 4.1. Interaction and Connectivity

The UHTM substrate must be designed to connect and interact with the EC via the bond coat (BC), and in case of failure of the latter the substrate must be able to survive for some time (time to be decided by regulating authorities in consultation with industry and engine manufacturers). Owing to the requirement for the UHTM substrate either to meet a specific property target or to have a balance of properties [7], and based on on-going research in our research group and other research groups—for example, see [35,44,46,54,55,56,57,58,59,60,61,62,63,64]—specific solutes should be used to make up the substrate’s chemical composition, namely, refractory metals (RMs), transition metals (TMs), simple metals (SMs), and metalloid elements (MEs) [4,5,6,7,25,35,44,45]. In the UHTM system, any individual interface (for example, any of the interfaces between the materials of the BC or the interface of the BC and substrate, see Figure 1) provides many different items of information that is distributed to (i.e., shared with) the whole system and processed.

The alloy design methodology NICE (a) recommends that (i) the substrate alloy should be in specific areas in parameter maps (parameters ΔH_mix_ (enthalpy of mixing), ΔS_mix_ (entropy of mixing), Δχ (based on electronegativity χ), δ (based on atomic size r), VEC (number of valence electrons per atom filled into the valence band), and Ω (=T_m_ΔS_mix_/|ΔH_mix_|)), for example, see Figures 1 and 2 in [7]) and that (ii) specific elements should be used, namely the RMs Mo, Nb, Ta, W, the TMs Cr, Hf, Ti, and the SMs and MEs Al, Ge, Si, Sn, and (b) indicates how element concentrations are selected [4,6].

The compatibility of the BC with the substrate and the desirable type of TGO (see Section 2) are essential to ensure interaction and connectivity. NICE directs us to select BC alloys from the Al–Hf–Nb–Si–Ti, Al–Cr–Nb–Si–Ti and Al–Cr–Hf–Nb systems [26,65,66,67] with specific phases in their microstructures using parameter maps (see Figure 13 in [65]) or solute maps (see Figure 13 in [67]). Additionally, NICE warns us about the avoidance of specific elements in BC alloys [68]. Furthermore, NICE (a) gives advice about (i) alloy compositions that can produce layered cast structures, (ii) about the desirable phases for such structures, and (iii) about avoiding liquation in BC alloys and (b) can recommend the chemical compositions of RCCAs or RHEAs for multilayer BC (see Section 5.5.1 in [67]) (note that RHEAs of the Al–Hf–Nb–Si–Ti system can have coefficients of thermal expansion that are compatible with RM(Nb)ICs and RCCAs/RM(Nb)ICs [7,66,67]).

**Figure 2:**
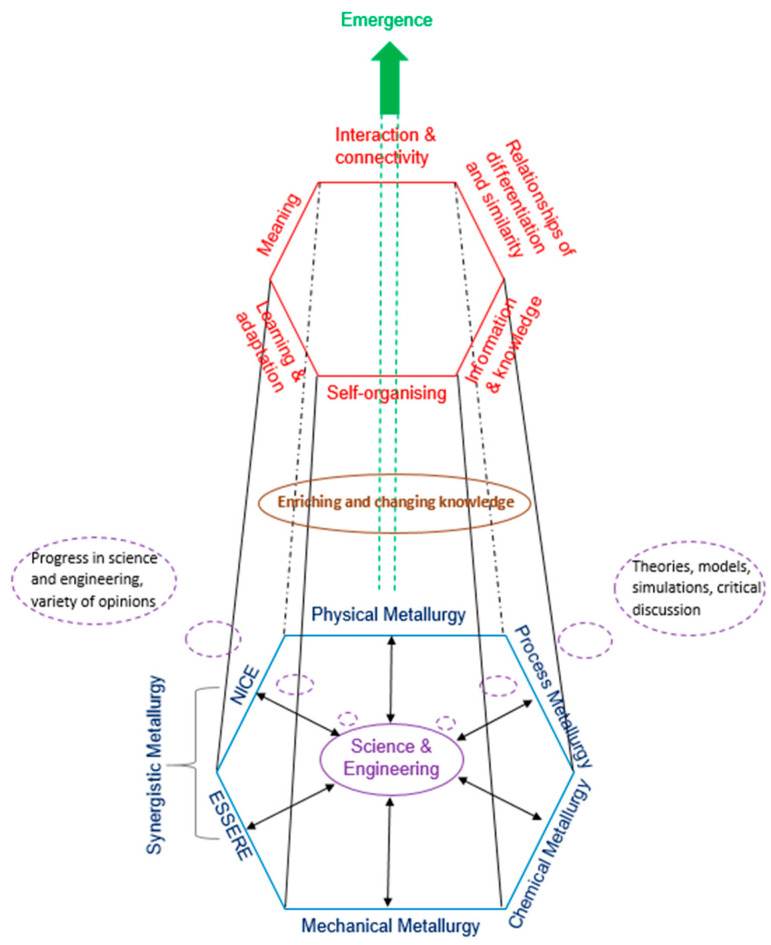
A schema of emergence for a metallic UHTM system (“the tower of emergence”). At the bottom is metallurgy with other science and engineering disciples (see text). At the top is complexity (see text). In the middle is enriching and changing knowledge (see text). For synergistic metallurgy see Sections 7 and 8 and Table 1 in [6], and the text. Science and engineering count on (i) theories, models, simulations and critical discussion (critical discussion is instrument of progress; what are the consequences of a theory, a model, a simulation? are the latter acceptable?) and (ii) progress in science and engineering and variety of opinions. The environment is key to emergence (see text, and Sections 7 and 8 in [6]). At all levels new structures arise from within the structure (see text and [6]).

For example, owing to interdiffusion between substrate and BC, the interaction and connectivity of these materials results in changes of their solute concentrations near interfaces and in the bulk, and to changes of properties of alloys and their phases. NICE points to what the accompanying changes would be and suggests how alloy design could handle the evolving situation. According to NICE, the aforementioned changes mean that (a) the position of each material shifts in parameter maps (see Figures 1 and 2 in [69], and Figures 12 and 13 in [65]) and also that (b) the positions of phases shift in parameter maps; for example, for the solid solution see Figures 3–5 in [69] and Figures 2a and 6a in [52]. Additionally, NICE tells us how, owing to changes in element concentrations, the properties of an alloy and its phases change; for example, for the substrate see Figure 2b in [7], Figure 15a in [39], Figure 15 in [40], Figure 21 in [41], and Figure 16 in [29], and for the Nb_5_Si_3_ silicide see Figure 5 in [70], and Figure 5a,b in [7].

Moreover, NICE shows how changes in solute concentrations can result in a change of (a) the phases that co-exist in the microstructure of the alloy, which can be “conventional” or high entropy (HE) or complex concentrated (or compositionally complex (CC)) phases [6] (for example, see Figure 29 in [20]) and of (b) the relationships between such phases (for example, see Figure 6b in [52]) and between the phases and the alloy (for example, see Figure 6 in [70]), and thus how the properties of phases change with each solute concentration (see Tables 5 and 6 in [20], and Tables 11 and 12 in [21]).

As a result of interaction with the environment and connectivity between the parts of the UHTM system and the environment, contamination with interstitials is unavoidable. NICE advises us about the implications of such contamination (for example, for the properties of bcc solid solution, see Figures 12d and 14c in [52]) and about the measures that one can take to control contamination (for example, see Figure 11 in [52] and Figures 14–17 in [21]).

NICE requires that alloy design must consider processability [6]. For example, regarding the substrate, NICE informs us that there will be macrosegregation of solutes in arc melted or plasma melted and cast alloys (see Table 5 in [39], Tables 4–6 in [42], and Tables 4–6 in [40]) as well as in directionally solidified (DS) alloys, where the macrosegregation profile would be affected by imposed growth rate(s) [71]. Regarding the BC, NICE warns us about macrosegregation, which can lead to liquation phenomena [67,68], but also advises that macrosegregation can be used to promote the formation of layered structures (see [26,66,67,68]). Owing to the chemical compositions and desirable phases in BC alloys, new processing methods may be required to be developed to deposit such BCs on metallic UHTM substrates (meaning that the existing coating processes/technologies may prove inadequate for the coatings required for UHTM systems in an era beyond that of Ni superalloys).

The alloy design methodology NICE directs us to consider cost, energy, processing and raw material issues as well as risk, sustainability and recyclability issues [6], that is to say, it links interaction and connectivity with IRIS, CEMI and ETS (see Abbreviations, and [6]) and thus brings NICE and ESSERE [6] in partnership with process metallurgy and physical metallurgy (see Sections 3 and 7 in [6]). The notion of synergistic metallurgy is key to handling interaction and connectivity in the design of a UHTM system. In particular, IRIS links risk with complexity and indicates that we must consider complexity when we assess, evaluate and calculate risk.

### 4.2. Relationships of Differentiation and Similarity

The structure of the UHTM system described in Section 2 discloses that it is organised in accordance with reason (*κατά λόγον*, kata logon, see morphological in Appendix A), that the co-existence of parts, i.e., substrate, BC, TGO, TC, is rational, that it is reason that gives them their specific difference and establishes relationships of differentiation and similarity and the morphological organisation of the system.

For example, consider an RCCA substrate consisting of the elements Al, Cr, Ge, Hf, Mo, Nb, Si, Sn, Ti, W to be cast using directional solidification. NICE guides the substrate’s design (see [4,6], and Figure 29 in [20]), and warns that, at different growth rates, macrosegregation of solutes [71] can lead to differentiation (see Figure 29b,c in [20], and [71]) and similarity (see Figure 29b,c in [20], and [71]) in the directionally solidified bar. NICE also advises about (i) differences and similarities between phases (see the data about relationships between solutes in the phases of the alloys discussed in [20,21]), and about (ii) the differences and similarities of properties of phases and of the contributions that solutes make to property changes (see Tables 5 and 6 in [20] and Tables 11 and 12 in [21]), which (the differences) are dependent on the phases that co-exist in the microstructure.

As another example, consider a BC with a layered structure made up of RCCAs or RHEAs that consist of Al, Hf, Nb, Si, Ti. NICE guides the design of different RCCA parts of the multi-layer BC, warns that macrosegregation of solutes can lead to differentiation and similarity in the microstructure and likelihood of liquation, and suggests chemical compositions for RCCA layers (see Sections 5.5 and 5.5.1 in [67] and Figure 29b,c in [20]).

Furthermore, NICE advises that differentiation and similarity in the UHTM system must be established giving the appropriate amount of attention to (i) cost, energy, processing and raw material issues and to (ii) the links with IRIS, CEMI and ETS [6], and thus brings ESSERE [6] in partnership with chemical metallurgy, process metallurgy and physical metallurgy (see Section 7 in [6]). Thus, synergistic metallurgy is key in handling differentiation and similarity in a UHTM system.

### 4.3. Self-Organising

The UHTM system, because of its design and function, should be able to create new structure(s) as it responds or adjusts (see Appendix A for self-organisation, feedback, adaptation) to environmental conditions. For this to materialise, NICE advises the use of Ge and/or Sn as solutes in the substrate, where Sn and Ge enrich the near surface areas (see [2,33,34,40,43]), control the vol.% of the bcc solid solution in the substrate (see Figure 9 in [39], Section 6.2 in [42], and [20,21,40,47,50]), control the mass change in oxidation in the pest oxidation regime and at high temperatures (see Figure 16 in [39] and [40,49]), and control the chemical composition of phases (for the bcc solid solution, see Figures 9, 10, 14 and 18 in [21]; for the Nb_5_Si_3_ silicide, see Figure 16 in [21], Figure 13 in [42] and Figures 7–9 and 21 in [20]; for the C14-NbCr_2_ Laves phase, see Figures 3 and 4 in [20]; and for the A15-Nb_3_X compound (X = Al, Ge, Si, Sn), see Figures 11–13 and 19 in [20]). Regarding the BC, NICE indicates that changes in Al or Si solute concentration will “shift” the BC in parameter maps (see Figure 8 in [7]). The UHTM system should be able to organise in time from its own internal dynamics.

NICE advises that self-organisation in the UHTM system requires control of the chemical composition of elements like Ge and Sn in the substrate and Al and Si in the BC during the processing and manufacture of the UHTM system, and links self-organisation with IRIS, CEMI and ETS [6]. Thus, self-organisation brings NICE and ESSERE [6] in partnership with chemical metallurgy, process metallurgy and physical metallurgy. Synergistic metallurgy is key for self-organisation in a UHTM system.

### 4.4. Information and Knowledge

Information and knowledge about the different parts of a UHTM system, their phases and properties are essential for the system’s design and development. Information and knowledge together with the follow-on understanding and the feedback that operating UHTM systems produce as they evolve, are key to improving the properties of such systems. In this context, NICE, via its knowledge production and perspective of the subject [6], uses the intimate and inseparable connection of the signified with the signifier (see information in Appendix A). For example, chemical analysis data of a phase (signifier), say Table 1 in [21] or Table 1 in [20], connects with maps of phases, say Figure 13a in [21] and Figure 16 in [20] (signified), that link with relationships between solutes in phases, say Figure 13b–d in [21] and Figure 17 in [20] (signified) and with the contributions of solutes to property (Young’s modulus, nano-hardness) changes, say Tables 9 and 10 in [21], and Tables 5 and 6 in [20] (signified). The partnership of synergistic metallurgy with chemical, mechanical, physical and process metallurgy, and its working together with the science and engineering disciplines is essential to controlling the uncertainty of information and knowledge resulting from contamination and partiality.

### 4.5. Learning and Adaptation

The acquisition of knowledge and skills through study (experimental and theoretical) of the different parts of the UHTM system and their phases and properties to complement the design and function of the system is essential for its design, development, manufacture and in-service use. A significant part of the learning and adaptation exercise will be mathematical (see learning in Appendix A) in nature, confronting problems and problem situations about the material system with critical arguments to seek solutions. It will be concerned with the material system’s structures themselves and the functions of their parts, i.e., with the chemical composition of the materials that make up the structures of systems; with their “architecture” and functional, mechanical and physical properties; with evolutionary changes, which will be dependent and/or triggered by environmental conditions; and with the dependence upon or their adjustments to the environmental conditions. Adjustment/feedback from the parts of the structures to the behaviour of the system will be essential. Evolution will proceed largely probabilistically under changing problem situations. Solutions will create new problem situations.

NICE does this systematically, utilising existing knowledge and producing and discovering new knowledge using synergy, entanglement, self-regulation and synergistic metallurgy (see Section 7 in [6,20,21]). NICE regards the achieved results as the starting points for further exploration, for example, for substrate see, in chronological sequence, [20,21,39,40,53,72] or [33,40,50,53] and for BC see, in chronological sequence, [26,65,68] or [66,67]. The process of learning and adaptation contains in itself a “theory” (see theory in Appendix A and below) of emergence.

### 4.6. Meaning

The parts of a UHTM system, because of their solute elements and phases, via their interaction and connectivity, and through their dealing with the environment, are creators of useful and important, i.e., meaningful, relationships and generators of meaning. For example, the aforementioned (solutes, phases, etc.) produce meaning for the material system by enabling it to achieve its objectives.

The material system works in relation to its environment. The notion of meaning embeds and entangles the material system in its environment. The philosopher Wittgenstein treated meaning as a public matter. For Wittgenstein, meaning is use [73]. Additionally, meaning = concepts (complex and simple), see [74]. Materials or material systems generate meaning as they evolve. Evolution can change the performance of material systems, their quality and usefulness and practicality. Synergistic metallurgy helps the discovery of important new data and unlocks the imagination so as to produce new experiments and interesting new ideas—it generates meaning.

### 4.7. Emergence and Emergent Properties

Seen in the light of the above discussion, which was informed by NICE [4], its alloy design landscape and the notion of synergistic metallurgy [6], the life of a UHTM system, from the early stages of its design to the later stages of its in-service use, is inexorably linked with (i) problem-solving and (ii) the discovery of new facts and new possibilities. Both (i) and (ii) also benefit from NICE and synergistic metallurgy [6]. In all of the different ways of learning or of acquiring or of producing data, information, knowledge and understanding, not only will the environment bring change, it will also be us (meaning the communities using, designing, developing, and producing materials) who select and change the environment (for example, by changing aviation fuels) and the worldwide community, with its impact on the environment. It is not only the environment that changes us, it is also we who select and change the environment. We shall learn from the environment in being challenged by it. NICE and synergistic metallurgy assist the alloy designer in this learning exercise, see [6].

The properties and behaviours of a UHTM system are emergent. Emergence is shown schematically in Figure 2. The latter portrays what I call the tower of emergence, which consists of three parts. In the bottom we have the interaction of metallurgy with other science and engineering disciplines—for example, chemistry, geology, mathematics, physics and chemical, computing, mechanical, mining and mineral processing, and systems engineering. In the top we have interaction and connectivity, relationships of differentiation and similarity, self-organising, information and knowledge, learning and adaptation, and meaning, which delineate complexity and the ascending emergence. Data, knowledge and understanding, resulting from emergence, update all three parts of the tower of emergence. In Figure 2, synergistic metallurgy encompasses NICE and ESSERE (see Sections 7 and 8 and Table 1 in [6]). NICE has demonstrated the coupling of phases, or of alloy and phases (with or without contamination by interstitials) in RM(Nb)ICs and RCCAs/RM(Nb)ICs or RHEAs/RM(Nb)ICs via the parameters Δχ, δ and VEC (for example, see Figures 6, 10, 14, 20e, 23a and 24a in [20]; Figures 9h and 12c,d in [21]; Figure 6 in [70]; Figures 3a,b and 6a–c in [52]; and, for contaminated with oxygen phases, see Figure 15d–f in [21] and Figure 10 in [52]). Instead of NICE, other approaches or proposals or methodologies of alloy design could be used by researchers, but with the proviso that they connect with risk, material/environment interactions and evolution, as depicted by ESSERE, see Section 7 in [6].

In the middle of Figure 2 we have the enrichment and change of knowledge that results from the teamwork of engineering and sciences with the humanities and arts (e.g., philosophy, history, literature) for solving problems in a spirit of collaboration that pays attention to the pluralism of traditions and values. The collaboration of engineering and sciences with the humanities and arts is (in my opinion) *sine qua non* for succeeding in a rapidly changing world and for facilitating a deeper intellectual understanding and use of materials. I hinted on this in the discussion in the preceding sections, in various entries in Appendix A (for example, see information, knowledge, learning, meaning, morphological, problem, theory) and shall do the same below. Let us not forget that much of what we would today call science was once called natural philosophy. While science can supply knowledge of means, it is for philosophy to discuss the choice of fundamental ends [75]. Ordinary perception involves a joint operation of the imagination and the understanding [76]. Art in its freedom succeeds in uniting form and matter [77].

The above discussion about UHTM systems shows that the well-articulated and familiar practice regarding the design of environmental coatings for engineering components based on state of the art Ni-based superalloys is challenged by a practice of a different kind that can interact with it. We have a new practise developing from another practise and we perceive a change. The latter is accompanied by changing degrees of awareness on the part of participants.

Regarding emergent properties and behaviours, we need to consider their measurement and calculation and the role of observer and practitioner/specialist. Measurements are themselves physical processes and we must not forget Einstein’s warning that “not everything that can be counted counts, and not everything that counts can be counted.” In the context of this paper, observer means the person who wants to know what is occurring and, for example, asks “should I take it seriously?,” “should I simply continue as before?,” and practitioner/specialist means the dedicated professional who wants to know what to do and what needs to be done. The observer can be a research manager or a director of a research centre, or someone whose job description touches the funding of research in progress, or a researcher who is looking for opportunities to diversify (broaden) his/her research. The practitioner/specialist is a metallurgist or metallurgy team who design(s) and develop(s) UHTM systems, or an engineer or engineering team(s) that design(s) structure(s) or “engineering platforms” that make use of such systems, or engineer(s) engaged with the manufacture and in-service life and repairs of such systems, or a researcher whose research focus is on alloy design, or a research team whose research priorities include topics that support alloy design and development. The enquiries of observer(s) must take into account the concerns of practitioners/specialists. The practitioners/specialists must pay attention cautiously to what observers have to say. The NICE alloy design landscape shows how this could be done [6].

To this discussion I wish to add here a few more remarks. On the subject of gathering and interpretation of information for a UHTM system using measurements, the key issues are (i) which conditions make the measurement possible and reliable; (ii) would measurements involve the use of an “ideal” UHTM system; (iii) which mathematical relations among measurement numbers would be empirically significant (e.g., different measurement scales convey different kinds of empirically significant information); (iv) the measurement of parameters to be used in model(s) to calculate the emergent; (v) what would be the interaction between the system (as a whole or its parts separately), instrument(s), the environment (which includes the measuring subject(s)) and the calibration process; and (vi) the theoretical or statistical model (i.e., a representation constructed from simplifying assumptions) used.

Experimental results would be expressed in terms that result from the application of statistics. Their explanations would call upon statistical concepts that are developed within frameworks that make assumptions about the meaning of concepts such as probability, which is a matter of significant philosophical argument. Models of probabilistic causation need to guard against the possibility that probabilistic assumptions between events may be spurious rather than genuinely causal.

Data would be subjected to statistical analysis, with assumptions about the shape of the distribution and the randomness of the effects of the environment, to give the measurement outcomes. The latter should be corrected for systematic effects. Corrections would be based on assumptions about how the instrument(s) work and how they interact with the whole system or its parts separately, and with the environment. The calibration would involve additional assumptions about the instrument(s), the calibrating apparatus(es), the quantity(ies) being measured and the properties of measurement standards (the uncertainty associated with a measured outcome is connected with calibration of the instrument(s)).

In the light of the above discussion, we are confronted with some key questions. Can we comprehensively handle the phenomenon of emergence? Can we develop a theory of emergence to predict properties, say mechanical, functional or environmental properties? Can we describe emergence with mathematical equation(s), for example as a function of interaction, connectivity, information, self-organization, meaning etc.? Trying to interpret or to understand a theory of emergence or mathematical equation(s) about emergence will in fact be raising a problem of understanding, i.e., a higher-level problem.

It is clear that emergence has quantitative and qualitative inputs. We know from the Nobel laureate Bertrand Russell that “physics is mathematical not because we know so much about the world but because we know so little; it is only its mathematical properties that we can discover” [78]. Additionally, we know from the Nobel laureate Richard Feynman that “the next great awakening of human intellect may well produce a method of understanding the qualitative content of equations” [79]. Moreover, according to the philosopher of science Paul Feyerabend “no single theory ever agrees with all the known facts in its domain.” “(We can) distinguish two different kinds of disagreement between theory and fact: numerical disagreement and qualitative failures. The first case is quite familiar: a theory makes a certain numerical prediction and the value that is actually obtained differs from the prediction made by more than the margin of error.” “The second case, the case of qualitative failures, is less familiar, but of much greater interest. *Ad hoc* approximations conceal, and even entirely eliminate, qualitative difficulties. They create a false impression of the excellence of our science.” “Whenever we select our evidence in an unprejudiced manner we find that theories fail adequately to reproduce certain quantitative results, and they are qualitatively incompetent to a surprising degree” (parenthesis and underlining mine) [17] (*Ad hoc* explanations are not independently testable (meaning independently of the effect to be explained)).

The emergent appears and is thought, by which I mean (a) that it is observable using instrumentation to observe, record, measure and collect data, and (b) that it is looked at, considered, speculated, and theorized. If we were to use the equivalent Greek words, the emergent *phainetai* (*φαίνεται*, appears) and *noeitai* (νοείται, is thought); this should not be confused with Kant’s phainomena (φαινόμενα) and noumena (νοούμενα) [77] (see also [80], for a critique of Kant’s use of the word nooumenon (νοούμενον). Ancient Greek philosophers also distinguished between phainomenon and nooumenon, for example Anaxagoras opposed what is thought to what appears).

A quantitative account of emergence, if it were to be possible, will inevitably lack qualities but a comprehensive one, if it were possible, must incorporate qualities. It is hard to see how the assertion “all is number” could apply in the case of emergence. Does this mean that we should quit depicting emergence in a UHTM system mathematically? Designers of materials systems and designers of engineering structures need numerical values of properties, i.e., they need numbers, to design, but they must be aware of the limitations. But who can be expected to predict the future? The apparent impossibility should not stop researchers from trying. For example, we could test short-term analyses of the problem that was presented at the start of this paper against long-term ones, while simultaneously subjecting long-term positions or convictions to shorter-term challenges. Once in a while a door opens and allows the future in [81].

## 5. Conclusions and Future Prospects

Conclusions: Emergence is a characteristic feature of a UHTM system. Its properties and behaviour, for example, mechanical or functional properties and oxidation, are emergent. Emergence results from the way in which synergistic metallurgy and other branches of metallurgy, engineering and science are connected and affect each other, affect material–environment interactions and affect complexity. Distinctive features of the latter, which is also entangled with the environment, are interaction and connectivity, relationships of differentiation and similarity, self-organising, information and knowledge, learning and adaptation and meaning. A quantitative account of emergence is unlikely to agree with all of the facts in its domain. The qualitative content of emergence should not be ignored.

Future prospects: This paper has emphasised the importance of material–environment interactions in the design and development of UHTM systems. Prospects are good regarding research on substrate selection and on bond coat structure but more concerted effort is anticipated vis-à-vis the selection of top coat materials and UHTM system–CMAS interaction. The aforementioned are intricately linked with processability and sustainability matters, in particular the manufacturing of UHTM systems, a possible transformative shift in coatings technologies, and a focus on circular economy principles.

## Data Availability

The original contributions presented in this study are included in the article. Further inquiries can be directed to the corresponding author.

## Abbreviations

The following abbreviations are used in this manuscript:

BC	Bond coat
CEMI	Correlative environment material interactions (see Sections 3 and 7 in [6])
CC	Complex concentrated (also compositionally complex)
DS	Directional solidification
EC	Environmental coating
ESSERE	Entanglement, synergy and self-regulation (see Sections 3 and 7 in [6])
ETS	Evolution through survival (see Section 7 in [6])
HE	High entropy
HPT	High-pressure turbine
HTM	High temperature material
IRIS	Interwoven risk (see Sections 3 and 7 in [6])
ME	Metalloid element
NICE	Niobium intermetallic composite elaboration
RM	Refractory metal
RMIC	Refractory metal intermetallic composite
RHEA	Refractory metal high entropy alloy
RCCA	Refractory metal complex concentrated alloy
RMIC/RHEA	RMIC that also meets the definition of RHEA
RMIC/RCCA	RMIC that also meets the definition of RHEA
RHEA/RMIC	RHEA that is also a RMIC
RCCA/RMIC	RCCA that is also a RMIC
RM(Nb)IC	Refractory metal intermetallic composite based on Nb
RM(Nb)IC/RCCA	RM(Nb)IC that also meets the definition of RCCA
RM(Nb)IC/RHEA	RM(Nb)IC that also meets the definition of RHEA
RHEA/RM(Nb)IC	RHEA that is also a RM(Nb)IC
RCCA/RM(Nb)IC	RCCA that is also a RM(Nb)IC
SM	Simple metal
SX	Single crystal
TC	Top coat
TET	Turbine entry temperature
TGO	Thermally grown oxide
TM	Transition metal
UHTM	Ultra-high-temperature material

## Appendix A

The etymology of some terms used in the paper is given in order to help the reader to appreciate their importance for the approach to design that which was discussed in the paper.

Adaptation: The process by which a system becomes suited or adjusted to its environment or new conditions. The process of changing to suit different conditions [82], adjustment to environmental conditions [83], the act of changing something to make it suitable for a new situation [84].

Commensurability: Scientific theories are said to be commensurable when they can be discussed and compared using the same standards of measurement to determine which is more accurate/useful for solving specific problem(s).

Complexity: From complex (adj.) + -ity. Complex comes from the Latin complexus, past participle of complecti, which means “to entwine (interweave) around,” “to embrace,” a word that is based in part on the word plectere (from the Greek word πλέκω (pleko), meaning “to weave,” “to twist,” “to plait,” “to tangle,” “to braid” (interweave)). -ity is a word-forming element making nouns from adjectives and meaning (in this case) “condition or quality of being complex.”

The Greek word for complex is περίπλοκο (periploko), from περί (peri (about, concerning)) + πλέκω (pleko (knit)). The Greek word for complexity is περιπλοκότητα (periplokotita) or concerning knitted/interwoven things. A knitted item (πλεκτό, plekto) has structural complexity.

On account of its origin, the word complexity is linked with structure. Synergy, entanglement and self-regulation (see below) are measures of the structure of complexity of metallic UHTMs and UHTM systems. Structural complexity emerges from systems that display morphological organisation (see below).

Feedback: The modification or adjustment of a system by a result or effect of a process [85]; the modification or control of a process or system by its results or effects; reaction to a process or activity or the information obtained from such a reaction, process or activity [82]; reaction or response to a particular process; evaluative information derived from a response to a process [84]; information returned to a system [86].

Framework: A framework is a supporting structure around which something can be built; a system of rules, ideas, or beliefs that is used to plan or decide something [82]. A framework provides a set of assumptions, concepts, values and practices [87]. Frameworks populate the scientist’s world with a set of conceptual objects and (non-causal) relationships among them [88]. A framework can be thought of as consisting of a dominant theory together with what one might call a way of viewing things in tune with the dominant theory [18]. A framework is a set of basic assumptions or fundamental principles, that is to say, an intellectual framework [89]. There are frameworks of thought which are incommensurable [17,18].

Incommensurability: Incommensurability is closely connected with the question of the rationality of science and critical rationalism [15,17,18,89]. “In the natural sciences criticism is connected with experiment and observation.” “Rational discussion consists in the attempt to criticize, not in the attempt to prove or to make probable.” “Incommensurable frameworks and incommensurable concepts may exhibit many structural similarities.” “There exist scientific theories which are mutually incommensurable though they apparently deal ‘with the same subject matter’” [17]. Different scientific theories are often incommensurable in the sense that there is no theory-neutral body of observational judgments to arbitrate (decide, adjudicate) between them.

Information: Information is an abstract concept that refers to something which has the power to inform. The origin of the word is from the Latin word informatio (conception, teaching, creation), from the verb informare—inform (to describe, shape, fashion), from in-‘into’ + forma ‘a form’.

According to the Oxford English Dictionary [85], information is knowledge communicated concerning some particular fact, subject or event or is contrasted with data (i.e., obtained by the processing of data) or the action of imparting the knowledge of a fact or occurrence.

Information is constrained by the data and can be contaminated with error. There is intrinsic uncertainty to information owing to contamination and partiality.

The Greek word for information is πληροφορία (plirophoria) from the verb φέρω (phero), frequentative of pherein (φέρειν), which means to carry through, bring, carry, and πλήρης (pliris), which means full, complete. Πληροφορία literally means “conveys fully.” (A frequentative form of a word is one that indicates repeated action). The use of the word πληροφορία (plirophoria) can be traced back to Socrates, Plato, and Aristotle.

With plirophoria (πληροφορία) there is distinction of the signifier (σημαίνον (simenon)), i.e., anything that signifies, e.g., words on a page, an image of a microstructure, a table of chemical analysis data of a phase, and the signified (σημαινόμενο (simenomenon)), i.e., the concept that the signifier refers to. There is an intimate and inseparable connection of the signified with the signifier.

Knowledge: Knowledge is the state of knowing about a particular fact or situation. It is also the information, understanding and skills that one gains through education or experience, the state of being aware or informed, a branch of learning, a science, and an art [85].

The growth of knowledge consists in correcting earlier knowledge. Rational critical discussion and rational criticism are engines of the growth of knowledge. Our tentative solutions to problems (see below) are relative to our problems, and our problems are relative to the state of our knowledge. There may be much in the temporary state of our knowledge that is in error (i.e., mistaken). Variety of opinion is necessary for objective knowledge.

“For Plato, in the place of knowledge (episteme (επιστήμη) = scientia = science) are the plausible but uncertain and prejudiced opinions (doxa (δόξα)).” With this distinction Plato claimed that “all knowledge of the visible world consists of doxa, that it is tainted by uncertainty even if it utilizes the episteme. In this sense, all theories are and remain hypotheses: they are conjecture (doxa) as opposed to indubitable knowledge (episteme)” [89].

For Albert Einstein “information is not knowledge” and for Charles Kettering, American engineer and inventor, “knowledge is not understanding.” The sequence data–information–knowledge–understanding is not “crystal clear” (for example, there is “overlap” in the use of the words information and knowledge).

Constraints on the pursuit of knowledge can come from the methodology of knowledge production. Knowledge is also constrained by perspective of the subject. Culture affects how people share knowledge. Enriching and changing knowledge through the arts is a fruitful enterprise.

Learning: The acquisition of knowledge or skills through study, experience, or being taught [85]; the activity of obtaining knowledge; knowledge or a piece of information obtained by study or experience [82].

The Greek word for learning is μάθηση (mathisi), from the verb μανθάνω (manthano) of which the modern Greek equivalent is the verb μαθαίνω (mathaino), both of which mean “to learn.” The word μάθημα (mathima), which means “what one learns,” “what one gets to know,” is derived from the verb μανθάνω (manthano). The word mathematics (μαθηματικά) comes from the Greek word μάθημα (mathima).

According to Plato, mathematics is concerned with special epistemological problems. Aristotle defined mathematics as “the science of quantity.” The mathematician Benjamin Peirce defined mathematics as “the science that draws necessary conclusions.”

Rationalists such as Descartes placed emphasis on the importance of mathematics for our understanding of the world, whereas empiricists such as Berkley, Hume, and Locke disparaged it. For both the rationalists and empiricists the objects of mathematics were taken to be our ideas.

Descartes vision was of a wholly a priori science. “Hume’s attitude was that our knowledge of mathematics, so far as it is genuine, is knowledge of ‘relations between ideas’, and this is to be contrasted with knowledge of ‘matters of fact and existence,’” which interested him. “Newton did not suppose that his laws of motion or his law of gravitation could claim any a priori status” and cited experiment and observation in their support [90], see also [91,92].

Meaning (νόημα (noema) in Greek, mental object [93]): There is no consensus on exactly what a noema is.

According to the Cambridge Dictionary [82], if something has meaning it has a purpose, and is worthwhile, it is of significant quality, of importance or value or represents or expresses something.

According to Husserl [94] noema exemplifies (stands for, implies) the object or content of a thought or judgement or perception. According to Sokolowski [95], noema appears to be (seems to be) what is intended by acts of perception or judgement, whether it be a material object, a picture, a word, a mathematical entity, another person precisely as being perceived, judged or otherwise thought about. According to Gurwitsch [96], most things can be perceived in different ways, and from different perspectives, what is perceived in such an act is a noema (meaning) and the thing itself is understood as the collection of noemata (meanings) associated with it. According to Morehouse [97], meaning is a relationship between two sorts of things: signs and the kinds of things they intend, express, or signify. In other words, meaning is the relationship between signifier and signified (see information above).

Metaphor: From the Greek word μεταφορά (metaphora) which is derived from the Greek verb μεταφέρω (metaphero). The word means to transfer (“to carry over”) a word to a new sense. Mεταφέρω (metaphero) is a combination of the words μετά (meta), which indicates change, and the verb φέρω (phero), which means “to bear, to carry, to transfer.”

Morphological: From the Greek word μορφολογία (morphologia)—itself from μορφή (morphi), which means form, outward appearance, structure, shape—plus λόγος (logos) derived from the Greek verb λέγω (lego), which means to tell, say, speak (thus logos means word, reason). Therefore, morphology could be understood as study relating to the form or structure of things.

Heraclitus was the first who used logos (λόγος) for a principle of order and knowledge. For Heraclitus, logos provided the link between ratio and discourse and rational structure of the world.

For the ancient Greeks the mode by which the universe (cosmos (κόσμος)) was governed was found κατά λόγον (kata logon), i.e., was found in relations in accordance with reason that governed the coexistence of things. Reason endows (provides, supplies) sensible things (things perceived by the senses) with form (morphi), it gives them their specific difference. Consequently, it establishes relationships of differentiation and similarity.

Organisation: the way in which something is undertaken or arranged, arrangement according to a particular system [82].

The origin of the word organisation is from the verb to organise and the noun organ, which is from the Latin organum, itself from the Greek organon (όργανον), which derives from the verb οργανώνω (organono) from οργαν(όω) + ώνω, which means accomplish something with due consideration (i.e., with the amount of attention that should be given owing to its importance, or having given thought upon reflection).

Problem: something that requires thought and skill for resolution, difficulty calling for a solution or is a cause of concern, a question raised for inquiry, consideration, or solution [83]. “Ernst Mach’s philosophy was that any method, any type of knowledge could enter the discussion of a particular problem” [14] (Ernst Mach was a physicist and philosopher who contributed to the physics of shock waves).

Self-regulation is the ability to control behaviour in the pursuit of goals/targets. In the alloy design methodology NICE, self-regulation is linked with the oxidation goal/target [6].

Self-organisation is a process by which a system adjusts through internal interactions allowing the system to learn or create new structure. Feedback (see above) and adaptation (see above) contribute to self-organisation.

Standards: “Standards (rules) determine the structure of research in advance, they guarantee its objectivity, (and) they guarantee what we are dealing with rational, scientific action.” (On the other hand), “every action and every piece of research can be regarded both as a potential instance of the application of rules and as a test case” [14] (parentheses mine).

“Strung together” is a phrasal verb. It means to combine things into a whole or to create something by putting things together.

Theory: The word derives from the Greek word θεωρία (theoria), which in ancient Greece meant “looking at,” and is from the verb θεωρείν (theorein), which means “to consider, speculate, look at.”

Modern uses of the word theory: According to the Collins Dictionary [84] a theory is a formal idea or set of ideas that is intended to explain something. According to the Britannica Dictionary [86] a theory is an idea or set of ideas that is intended to explain facts or events. According to the Cambridge Dictionary [82] a theory is a formal statement of the rules on which a subject of study is based or of ideas that are suggested to explain a fact or event or, more generally, an opinion or explanation. According to the United States National Academy of Sciences, “theory” refers to a comprehensive explanation of some aspect of nature that is supported by a vast body of evidence [98]. According to the American Association for the Advancement of Science a “scientific theory” is a well-substantiated explanation of some aspect of the natural world, based on a body of facts that have been repeatedly confirmed through observation and experiment [99].

A theory may be inconsistent with the evidence not because it is incorrect but because the evidence is contaminated. Observations should be taken for granted after the most careful examination of their reliability [17].

We cannot really understand a scientific theory or the development/evolution of a class of materials without understanding its history [6]. To understand a theory, first, we need to understand the problem situation in which it arises. “The material which a scientist actually has at his disposal (e.g., laws, experimental results, mathematical techniques, epistemological prejudices) is indeterminate in many ways, ambiguous, and never fully separated from the historical background. This material is always contaminated by principles which s/he does not know and which, if known, would be extremely hard to test” [17].
